# Updating of HIMAC beam delivery with broad beam method

**DOI:** 10.1093/jrr/rrt158

**Published:** 2014-03

**Authors:** Manabu Mizota, Shigekazu Fukuda

**Affiliations:** National Institute of Radiological Sciences, Anagawa 4-9-1, Inage-ku, Chiba 263-8555, Japan

**Keywords:** HIMAC, heavy ion radiotherapy, broad beam method, beam energy

## Abstract

Carbon ion radiotherapy at HIMAC started in 1994 using two horizontal and two vertical beam ports with broad beam method. The maximum beam energy extracted from accelerators was 400 MeV/n for the horizontal beam port, and 350 MeV/n for the vertical one. In recent years, the scope of diseases that can be treated by the carbon ion radiotherapy has widened and their tumor targets have varied in dimension and location. So, situations of shortage in the beam range in treatment planning have increased.

In order to provide sufficient dose to the deeply seated target, we adopted higher energy beam, that is, 430 and 400 MeV/n for the horizontal port and the vertical port, respectively. For the introduction of the new energy beams, we carried out the following.

(1) Adjustment of new parameter-sets of the accelerators for the high-energy beams.

(2) Replacement of ridge filters with the redesigned ones for the high-energy beams.

(3) Updating irradiation condition tables in the irradiation control system and registering new beam data in the treatment planning system, on the base of dose distribution measurements.

As a result, the beam range for horizontal port and vertical port was extended ∼30 and 50 mm, respectively. This allows us to treat all patients without ingenuity of patient's posturing and beam weight for each direction.

On the other hand, adding new operational energies leads to a decrease in whole throughput, because it takes time to switch among the beam energies. We have at the same time simplified the procedures for the dose calibration and beam adjustment in the switching of energy.

The broad beam method has reached maturity as a standard irradiation method of carbon ion radiotherapy and has achieved superior clinical results. We will continue to make upgrades this system with maintenance of its performance and improve the usability of this systems.

